# Kisspeptin-neuron control of LH pulsatility and ovulation

**DOI:** 10.3389/fendo.2022.951938

**Published:** 2022-11-21

**Authors:** Harvey Stevenson, Samuel Bartram, Mikaela Maria Charalambides, Sruthi Murthy, Theo Petitt, Anjali Pradeep, Owen Vineall, Ikenna Abaraonye, Amelia Lancaster, Kanyada Koysombat, Bijal Patel, Ali Abbara

**Affiliations:** Section of Investigative Medicine, Department of Metabolism, Digestion and Reproduction, Imperial College School of Medicine, Imperial College London, London, United Kingdom

**Keywords:** kisspeptin, ovulation, oestrogen receptor, KNDy neurons, arcuate nucleus, anteroventral periventricular nucleus, gonadotrophin releasing hormone, luteinising hormone

## Abstract

Feedback from oestradiol (E2) plays a critical role in the regulation of major events in the physiological menstrual cycle including the release of gonadotrophins to stimulate follicular growth, and the mid-cycle luteinising hormone (LH) surge that leads to ovulation. E2 predominantly exerts its action *via* oestrogen receptor-alpha (ERα), however, as gonadotrophin releasing hormone (GnRH) neurons lack ERα, E2-feedback is posited to be indirectly mediated *via* upstream neurons. Kisspeptin (KP) is a neuropeptide expressed in hypothalamic KP-neurons that control GnRH secretion and plays a key role in the central mechanism regulating the hypothalamic-pituitary-gonadal (HPG) axis. In the rodent arcuate (ARC) nucleus, KP is co-expressed with Neurokinin B and Dynorphin; and thus, these neurons are termed ‘Kisspeptin-Neurokinin B-Dynorphin’ (KNDy) neurons. ARC KP-neurons function as the ‘GnRH pulse generator’ to regulate GnRH pulsatility, as well as mediating negative feedback from E2. A second KP neuronal population is present in the rostral periventricular area of the third ventricle (RP3V), which includes anteroventral periventricular (AVPV) nucleus and preoptic area neurons. These RP3V KP-neurons mediate positive feedback to induce the mid-cycle luteinising hormone (LH) surge and subsequent ovulation. Here, we describe the role of KP-neurons in these two regions in mediating this differential feedback from oestrogens. We conclude by considering reproductive diseases for which exploitation of these mechanisms could yield future therapies.

## Introduction

Major events in the physiological menstrual cycle including follicular development and ovulation are tightly regulated by intricate negative and positive feedback mechanisms in response to sex-steroids that underpin the hypothalamic-pituitary-gonadal (HPG) axis (1). Pulsatile secretion of hypothalamic gonadotrophin releasing hormone (GnRH) stimulates gonadotrophin secretion from the anterior pituitary gland, and subsequent folliculogenesis and oestradiol (E2) secretion from the ovaries (1). During follicular development, pulsatile GnRH secretion is modulated by negative feedback from circulating E2 (1). In contrast, at the preovulatory stage, high E2 concentrations exert positive feedback to result in the mid-cycle LH surge and ovulation (1). Both negative and positive feedback from E2 are mediated *via* oestrogen receptor-alpha (ERα) (2). Formerly, given the lack of ERα on GnRH neurons, the mechanism by which E2 exerts its feedback on GnRH neurons was unclear, but consistent with E2-feedback being mediated indirectly *via* upstream neurons (2). A putative mediator of this E2-feedback are hypothalamic neurons expressing the neuropeptide kisspeptin (KP).

KP is a key regulator of hypothalamic GnRH secretion and the HPG axis (3). Inactivating variants of the gene encoding KP (*KISS1*) or its receptor KISS1R (*KISS1R*) result in congenital hypogonadotrophic hypogonadism and a failure to proceed through puberty in humans and murine models (3–5). KP-neurons are located in two discrete hypothalamic neuronal populations in rodents; the arcuate nucleus (ARC) (which is equivalent to the infundibular nucleus in humans) and the rostral periventricular area of the third ventricle (RP3V) comprising of KP-neurons in the anteroventral periventricular (AVPV) nucleus and preoptic area (6). In particular, KP-neurons in the ARC (ARC KP-neurons) co-express Neurokinin B (NKB) and Dynorphin, and hence are termed Kisspeptin-Neurokinin B-Dynorphin (KNDy) neurons (7).

In this review, we explore evidence to support ARC KP-neurons as mediators of E2-induced negative feedback to regulate GnRH pulsatility, and RP3V KP-neurons as mediators of positive feedback in response to higher E2 levels to induce the mid-cycle LH surge/ovulation. The input of neuropeptides such as glutamate, as well as metabolic signals such as leptin are also considered. Finally, reproductive diseases that result in perturbation of LH secretion are considered, for which exploitation of these mechanisms may yield future therapies.

## Kisspeptin neurons and feedback by E2

The activity of kisspeptin neurons varies throughout the menstrual cycle and is tightly regulated by oestradiol (E2) (1). During the follicular and majority of the luteal phases of the menstrual cycle, the presence of low E2 results in inhibition of ARC KP-neurons (negative feedback) and thus maintains pulsatile secretion of GnRH (1, 2). In the mid-luteal phase, high E2 stimulates RP3V KP-neurons (positive feedback) which results in the GnRH/LH surge responsible for triggering ovulation (1, 2). This differential feedback response of KP-neurons by low and high E2 levels has been observed in multiple species. For instance, studies in rodents have demonstrated increased expression of *KISS1* mRNA levels in both the ARC during metestrus and dioestrus (low E2 state) and the RP3V during proestrus (high E2 state) (8). Furthermore, postmenopausal women (low E2 state) have increased *KISS1* mRNA levels in KP-neurons of the infundibular nucleus (equivalent to ARC in rodents) (9).

The mechanisms underlying the divergent feedback of E2 on KP-neurons are complex. ERα (encoded for by *Esr1*) is responsible for mediating both negative and positive feedback from E2 (10, 11). ERα can signal either by translocation to the nucleus and recruitment of cofactors to oestrogen response element (ERE) (classical pathway), or by recruitment of other transcription factors not *via* the ERE (non-classical pathway) (10, 11). Notably, E2-induced positive feedback occurs *via* the classical pathway, whereas negative feedback is mediated *via* the non-classical pathway (10, 11). In AVPV KP-neurons in the RP3V, E2 increases recruitment of ERα to the *Kiss1* promoter region which results in enhanced histone acetylation (12). In turn, histone acetylation induces chromatin loop formation between the *Kiss1* promoter and *Kiss1* gene enhancer, leading to an increase in AVPV-specific *Kiss1* gene expression (12). The opposite effect is observed in ARC KP-neurons whereby the *Kiss1* promoter region undergoes histone deacetylation and subsequent reduced gene expression following E2 (12).

A recent murine study shed light on how KP-neurons respond divergently to high and low E2 concentrations, by revealing differential RNA transcriptional responses between KP-neurons in the ARC and the RP3V (13). They identified 1583 oestrogen-responsive genes (majority suppressed) within the ARC, and 222 genes (majority upregulated) in the RP3V, thus showing that there are more oestrogen-responsive genes in the ARC than RP3V (13). Interestingly, whilst *Esr1* (which encodes ERα) expression was increased in both RP3V and ARC KP-neurons, no differences in *Esr2* (which encodes ERβ) or *Gper1* (which encodes G-protein coupled oestrogen receptor) were observed (13). Furthermore, ERα interacted with 8 of 70 E2-dependent transcription factors within the ARC, but 0 of 10 E2-dependent transcription factors within RP3V (13). Despite disparate E2-regulation, RP3V and ARC KP-neurons displayed 96 overlapping genes, with changes in 62 genes being analogous (e.g. *Ghsr, Pgr, Npr2, Gad2, Calm1, Pcp4*), and 34 genes being regulated in a contrasting manner (e.g. *Kiss1, Vgf, Chrna7, Tmem35a*) *(* 13). Overall, these data indicate that the effects of oestrogens are predominantly mediated by ERα in both the ARC and RP3V neurons, and that there are more oestrogen responsive genes in the ARC (mediating negative feedback) than in the RP3V (mediating positive feedback) (13).

**Figure 1:**
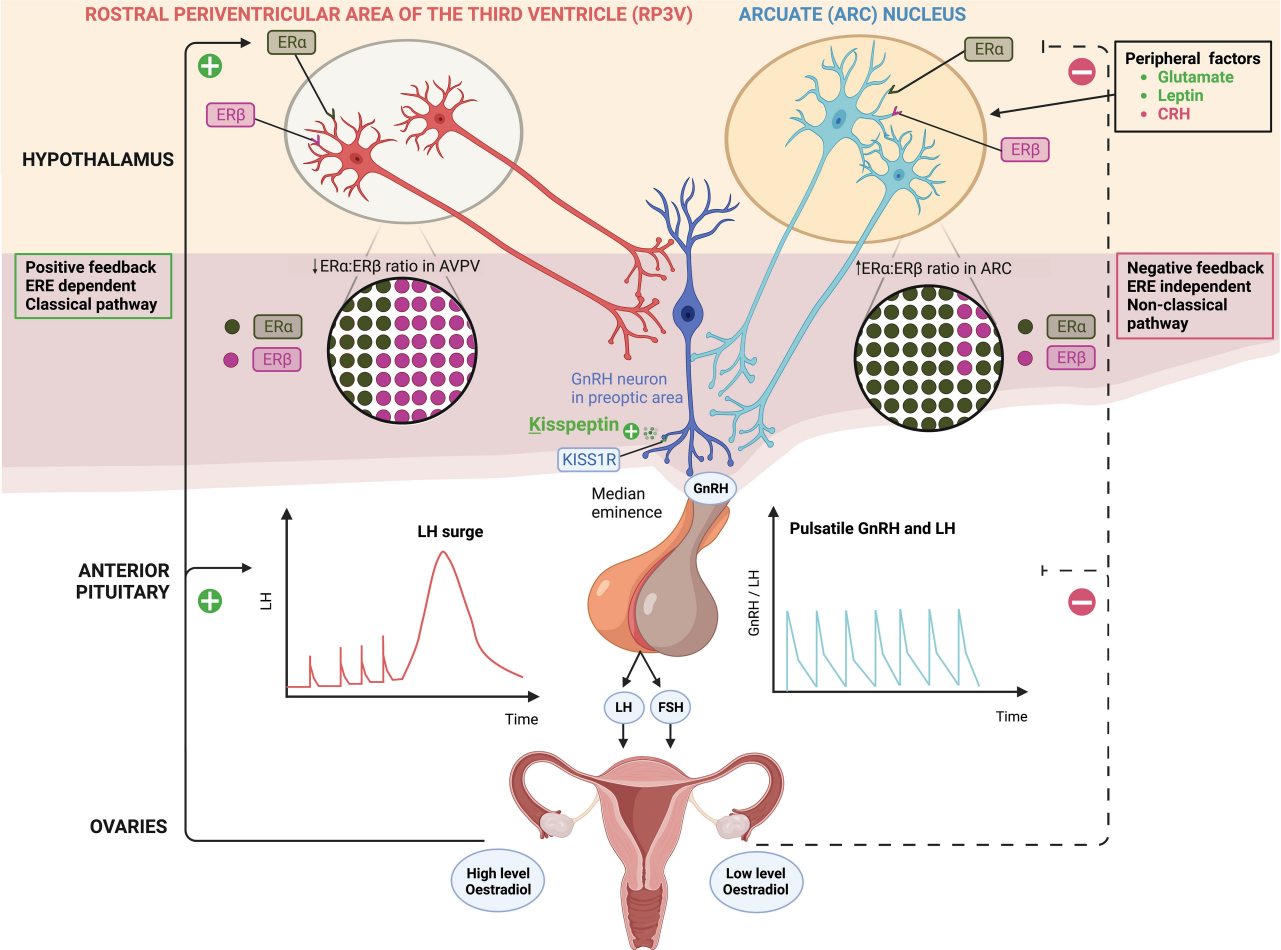
Distinct populations of kisspeptin neurons in the hypothalamus; kisspeptin neurons in the arcuate nucleus (ARC KP-neurons) and rostral periventricular area of the third ventricle (RP3V KP-neurons) which includes anteroventral periventricular (AVPV) nucleus. ARC KP-neurons exhibit episodic activity that induce pulsatile GnRH and LH secretion through oestradiol (E2) mediated negative-feedback and are known as the ‘GnRH pulse generator’ depicted on the right. Synchronisation of these neurons is driven by NKB and dynorphin; NKB stimulates whereas dynorphin inhibits kisspeptin release (not shown). Peripheral factors are depicted [glutamate (stimulatory), leptin (stimulatory) and CRH (inhibitory)] acting at the level of the brain to regulate kisspeptin output. By contrast RP3V KP-neurons induce an LH surge through E2 mediated positive-feedback and ovulation depicted on the left. Oestrogen receptor α (ERα) is responsible for mediating both negative and positive feedback from E2. ERα can signal either by translocation to the nucleus and recruitment of cofactors to oestrogen response element (ERE) (classical pathway), or by recruitment of other transcription factors not *via* the ERE (non-classical pathway). Notably, E2-induced positive feedback occurs *via* the classical pathway, whereas negative feedback is mediated *via* the non-classical pathway. Oestrogen receptor β (ERβ) has been shown to potentiate E2 positive feedback in the RP3V in the presence of high E2 levels and has an inhibitory effect on ERα in the AVPV in the presence of low E2 levels. Regardless of E2 levels, the ratio of ERα:ERβ is twice as high in the ARC as in the AVPV.ARC, arcuate nucleus; AVPV, anteroventral periventricular nucleus; CRH, corticotrophin-releasing hormone; E2, oestradiol; ERα, oestrogen receptor α; ERβ, oestrogen receptor β; ERE, oestrogen response element; FSH, follicle-stimulating hormone; GnRH, gonadotrophin releasing hormone; KISS1R, kisspeptin receptor; LH, luteinising hormone; NKB, neurokinin B; RP3V, rostral periventricular area of the third ventricle.

Whilst ERα is the major mediator of E2 feedback on kisspeptin neurons, ERβ has been shown to potentiate E2 positive feedback in the RP3V. For instance, OVX rats have a two-fold increase in the *Esr1/Esr2* ratio in the ARC compared to the RP3V, irrespective of E2 replacement, consistent with greater presence of ERβ in the RP3V (14). ERβ regulates E2-induced positive feedback by increasing transcriptional activity of ERα and enhancing the responsiveness of RP3V neurons to high concentrations of E2 (14). In the presence of low E2 levels, ERβ has an inhibitory effect on ERα in the RP3V and thus it could have an indirect role in mediating E2 negative feedback (14) (**Figure 1**).

The feedback mechanism of E2 is particularly intricate within ARC KP-neurons. KP-neurons in the ARC co-express Neurokinin B (NKB) and Dynorphin and hence are termed KNDy neurons. E2 modulates pulsatile KP release by adjusting the stimulatory (NKB) and inhibitory (dynorphin) inputs on ARC KNDy neurons (15, 16). Whilst E2 treatment reduces NKB gene expression, dynorphin (Pdyn) gene expression levels remain unchanged (15, 16). This suggests that the E2 negative feedback on ARC KP-neurons is predominantly mediated through NKB suppression.

## Kisspeptin neurons in arcuate nucleus regulate GnRH pulsatility

ARC KP-neurons regulate GnRH pulsatility, and are regarded as the ‘GnRH pulse generator’ (17). ARC KP-neurons innervate GnRH neurons at their distal dendrons in the median eminence of the hypothalamus and release KP (13). The activity of KP-neurons is episodic as indicated by studies measuring multiple unit activity (MUA) near ARC KP-neurons in goats (18, 19) and rats (20), as well as calcium activity in ARC KP-neurons in female mice (21). KP is the main output signal of ARC KNDy neurons to induce pulsatile GnRH, and in turn LH, secretion (22). Optogenetic studies reveal that activation of channel rhodopsin expressing ARC KP-neurons, induces pulsatile LH secretion in *Kiss1-Cre* mice; whereas inhibition of ARC KP-neurons suppresses pulsatile LH secretion (both frequency and amplitude) (22, 23). Indeed, global knockout of the *Kiss1* gene (*Kiss1^-/-^)* in ovariectomised female rats leads to complete loss of LH pulses (17, 24) and specifically, knock out of greater than 90% of ARC *Kiss1*-neurons results in marked suppression of LH pulses (17). Interestingly, rescuing a minimum of 20% of KNDy neurons is sufficient to restore pulsatile LH secretion (17). Collectively, these data suggest that ARC KP-neurons act as the intrinsic GnRH pulse generator and that there is redundancy in the number of ARC KP-neurons needed to maintain GnRH pulsatility.

### NKB and dynorphin modulate GnRH and LH pulsatility

Kisspeptin is co-expressed with NKB and Dynorphin in ARC KNDy neurons and this expression pattern is consistent across several species including the mouse (25), rat (24, 26), goat (19), monkey (9), and sheep (27). Whilst the output of ARC KNDy neurons is KP, the synchronisation of these neurons is driven by NKB and dynorphin. NKB initiates whereas dynorphin terminates KNDy neuronal activity in order to induce pulsatile secretion of KP (18, 19, 28, 29). The effects of NKB and dynorphin on ARC KP neuronal pulsatility are mediated *via* neurokinin-receptor 3 (NK3R) and κ-opioid receptor (KOR), respectively (19, 30).

Neurokinin B and NK3R agonists have been shown to stimulate ARC KP-neurons and downstream GnRH/LH pulsatile secretion in various species. For instance, in ovariectomised (OVX) goats, central administration of NKB (direct injection into the ARC) (31) as well as peripheral administration of NK3R agonist (intravenous injection of senktide) increases multiple-unit activity (MUA, a measure of GnRH/LH pulsatility) (19). Notably, serum LH levels remain unchanged in OVX goats with E2 replacement, thus indicating that NKB action on ARC-KP-neurons is more sensitive to low rather than high E2 concentrations (19). Similar findings have also been observed in OVX ewes (32) and mice (30); however adult rats demonstrate reduced LH pulse frequency following senktide, thus suggesting species variation (33, 34). LH pulse frequency is reduced following NK3R antagonists (32, 35), and specifically MRK-08 has been shown to lead to complete abolishment of LH pulses (27). Activation of KOR (by dynorphin/KOR agonists) decreases MUA frequency in OVX goats (18) and mice (30); whilst inhibition of KOR (by KOR antagonists: nor-binaltorphimine, PF-4455242, naloxone) increases MUA in OVX goats (18, 36), ewes (32), mice (37) but blocks MUA/LH pulses in OVX rats (34). Interestingly, in gonadal intact prepubertal rats, NKB-induced LH pulses are unaffected by Dynorphin (Dyn) but blocked by a KP receptor antagonist (38). This supports the notion that NKB related LH secretion is dependent on KP, but not on Dyn signalling (38). Overall, these data indicate that NKB activity is critical for the activation of ARC KP-neurons and generating the GnRH pulse.

### External factors and ARC-KNDy-neuronal firing

The frequency and amplitude of ARC KP neuronal firing is influenced by several positive and negative modulators in addition to E2 (39). Glutamate (24) and leptin (40) are positive modulators that have stimulatory effects on ARC KP-neurons. Glutamate induces LH pulses *via* KP-neurons as demonstrated in *Kiss1*-KO rats that fail to increase LH secretion following monosodium glutamate/NMDA (glutamatergic agonist) injections (24). Furthermore, optogenetic stimulation of ARC-KNDy neurons mitigates upstream glutamate-induced signalling, thus highlighting the importance of ARC-KP-neurons in mediating responses to glutamate (41).

Leptin, a key mediator of energy availability, modulates ARC KP neuronal firing frequency (40), although the action of leptin on KP-neurons is not thought to be direct (42). In leptin-resistant states (due to high leptin concentrations as may occur in obesity), ARC KP-neurons are quiescent inhibiting reproduction (40). Leptin-resistant mice have reduced ARC *Kiss1* expression, and are infertile (40). Thus, one mechanism by which obesity causes hypogonadism is *via* the induction of leptin resistance, leading to decreased action of KP-neurons *via* interneurons.

Padilla et al. explored how starvation can negatively modulate KNDy neuron firing by investigating Agouti-related peptide (AgRP) neurons, which project to ARC KP-neurons (43). AgRP is a neuropeptide that potently stimulates appetite and reduces energy expenditure in response to starvation (44). Ablation of AgRP neurons in neonatal mice resulted in less inhibitory input to ARC KP-neurons (43). Using optogenetic techniques, AgRP neurons were shown to inhibit both ARC KP-neurons and KP-expressing neurons in the AVPV (43). Using chemogenetic techniques, chronic AgRP signalling was shown to impair fertility in mice (43). Indeed, Wu et al. demonstrated that fertility is restored in leptin-deficient mice when AgRP neurons are ablated (44). Since there appears to be no direct signalling between AgRP and GnRH neurons (43), ARC KP-neurons could provide a credible link between nutrition and fertility.

Emotional stress can also impair fertility through GnRH suppression (45). Lin et al. demonstrate that the central amygdala (CeA) suppresses the GnRH pulse generator in female mice in response to psychogenic and immunological stressors (45).The amygdala releases corticotrophin-releasing hormone (CRH), subsequently activating the hypothalamic-pituitary-adrenal-axis, in response to stress (45). Kinsey-Jones et al. found that high CRH suppresses ARC KP-neurons, leading to reduced LH pulsatility (46). Whilst the CeA exerts inhibitory effects on ovulation, the medial amygdala (MeA) stimulates it, since lesioning of the MeA blocks ovulation, whilst stimulation advances the time of the LH surge (45). Together, these data highlight how *Kiss1* neurons can integrate signals to modulate GnRH pulsatility and impair fertility in times of stress or metabolic disturbance.

## Kisspeptin neurons in RP3V induce an LH surge to trigger ovulation

Kisspeptin neurons in RP3V expressing ERα are present across mammalian species and induce an LH surge through E2-mediated positive-feedback (26, 47). Elevated E2 levels can initiate a pre-ovulatory LH surge across all studied mammalian species (48), however the mechanism downstream of this signal varies between species, with the hypothalamus identified as the site of primary action in rodents and sheep, whereas the pituitary is of higher relative importance in primates (49). More recently, neural progesterone (P4) has been identified as a key player in the generation of LH surges downstream of E2 signalling pathway (50, 51). Total P4 receptor knockout studies saw the absence of an LH surge, and reduced c-Fos (a marker of neuronal activation) in RP3V KP-neurons. Reintroduction of P4 receptors solely in the AVPV re-established the LH surge (50, 51). *In vitro* studies highlighted that P4 also augments kisspeptin expression and release from RP3V KP-neurons neurons in conjunction with E2 (52). Together, astrocytes local to the AVPV seem to be the source of the P4, induced by E2-moderated positive-feedback (53). Pre-ovulatory levels of E2 upregulate Kiss1 expression *via* histone acetylation in the Kiss1 promoter region (11), with preoptic/AVPV Kiss1 neurons being fundamental to the generation of a pre-ovulatory LH surge in rodents (54).

Further to relaying positive steroidal feedback to GnRH neurons, RP3V KP-neurons also act as an integrative hub for neural afferents involved in ovulation (55). LH surges in mice occur consistently at midday of the proestrus stage of their oestrous cycle (56) suggesting that importance of circadian rhythm to the LH surge. Neurotracing studies reveal that the suprachiasmatic nucleus (SCN) provides an afferent input to RP3V KP-neurons neurons through vasopressin (57). Unilateral lesion studies in the SCN reveal that *Kiss1* mRNA expression in RP3V KP-neurons neurons are regulated by an oscillator in the dorsomedial SCN (58). As such the dorsomedial SCN regulates GnRH activity during ovulation through upregulating expression of kisspeptin in RP3V KP-neurons neurons. Whether there is circadian control over ovulation in humans remains unclear (59, 60) although it seems possible given that shift workers are at risk of reduced fertility and irregular menstrual cycles (61, 62). However, any effects of shift work on menstrual cycles could also be due to high-stress conditions, which as previously described inhibit activity of KP-neurons.

Additionally, ERα-expressing noradrenergic cell groups may be involved in LH surge generation through modulating KP release from AVPV neurons (63). It is known that noradrenaline facilitates LH surge generation (64, 65), however a link to KP has only recently been posited. Administration of an α1-blocker reduces *Kiss1* expression in the preoptic area (POA) and ultimately LH release, suggesting that noradrenaline release facilitates KP synthesis prior to an LH surge (66). Interestingly α1-receptors have not been identified on RP3V KP-neurons neurons (66), indicating that noradrenaline modulates KP-neurons upstream of the POA. Due to the expression of α1-receptors in the SCN (67), it has been proposed that noradrenaline modulates vasopressinergic inputs from the SCN to RP3V KP-neurons neurons to mediate KP release in the median eminence (68).

RP3V KP-neurons neurons receive afferents from ARC KP-neurons *via* glutamate suggesting that ARC KP-neurons play a role in LH surge generation in addition to their role in pulse generation (29). Furthermore, optogenetic stimulation can spontaneously provoke LH surges in an E2-dependent manner (68). In ARC KP-neurons, glutamate expression is increased by high E2 levels (69). Thus, E2 may positively feedback on the ARC to increase glutamatergic inputs to the AVPV at the time of ovulation to facilitate LH surge generation. To what extent glutamatergic inputs play a role in LH surge generation remains unclear; glutamatergic inputs to RP3V KP-neurons neurons from ARC KP-neurons are not essential to the LH surge, as knocking out glutamate in all KP-neurons had no effect on ability of mice to undergo oestrous cycles (69).

## Kisspeptin neurons may be implicated in disorders of reproduction

Hypogonadotrophic hypogonadism is a deficiency of hypothalamic GnRH leading to decreased adenohypophyseal secretion of LH and FSH, resulting in failure to undergo puberty and infertility (3, 4). Two groups almost simultaneously identified an autosomal recessive cause of hypogonadotrophic hypogonadism in consanguineous families, caused by inactivating variants in *KISS1R (* 3, 4). Seminara et al. developed a *Kiss1r-*deficient mouse-model, which had small prepubertal ovaries and absent follicular maturation, replicating the hypogonadotrophic hypogonadism phenotype observed in humans (4). Conversely, an autosomal dominant activating variant of the *KISS1R* results in precocious puberty caused by early maturation of the HPG-axis (70).

Functional hypothalamic amenorrhoea (FHA) is a common cause of amenorrhoea in women of reproductive age (71). It is caused by reduced GnRH pulsatility, leading to deficient LH pulsatility, in the absence of structural abnormality (71, 72). Weight-loss and intense exercise regimens limiting energy availability, as well as psychological stress are the most common causes of FHA (73). Changes in activity of hypothalamic ARC KP-neurons in relation to such inputs modulates the activity of GnRH pulsatility and subsequently fertility (43, 74). Restoring fertility requires reestablishment of gonadotrophin secretion, rather than by sex-steroid replacement alone, which can be achieved by administration of KP (72). Animal models suggest that undernutrition is associated with decreased hypothalamic *Kiss1* expression in the ARC, and thus conceptually, KP administration is an attractive approach to restore reproductive health. Unfortunately, stimulation of gonadotropin production was not sustained after high dose chronic stimulation in women, which was attributed to tachyphylaxis (72). In order for replacing KP to represent a future therapy for FHA, more developing treatment protocols that can induce sustained stimulation of the HPG axis is much needed.

Another common cause of secondary amenorrhoea is polycystic ovary syndrome (PCOS) (71). GnRH and LH pulse frequency are increased in PCOS, which results in increased ovarian androgen production (a cardinal feature of PCOS). Indeed, LH pulse frequency is useful for distinguishing between FHA and PCOS, as the two commonest pathological causes of secondary amenorrhoea (73). Possible mechanisms contributing to increased LH pulse frequency include loss of progesterone negative feedback (due to the opposing effects of androgens) and enhanced GABAergic drive to GnRH neurons (75–77). Indeed, LH production is increased in PNA mice subject to chemo- and opto-genetic activation of GABA neurons in the ARC, highlighting the stimulatory role of GABAergic neurons have on GnRH neurons (77). However, there is evidence that KNDy neuronal activity is key to the increased LH pulse frequency and amplitude seen in PCOS. A study using constant, chronic letrozole delivery to PCOS mouse models found that *Kiss* and *Tac2* (encoding NKB in rodent species) gene expression were strongly upregulated in the ARC (78). Further, *cfos* expression was upregulated in ARC KP-neurons, indicating greater neuronal activation (78). However, not all features of PCOS are replicated in animal models of PCOS, as a recent study using PNA mice did not show that KNDy neuronal firing frequency was increased (75).

Women with PCOS and oligomenorrhoea had an increase in circulating KP pulse frequency (79), and whilst pulses in circulating KP and LH levels were coupled in eumenorrheic women, this coupling was lost in oligomenorrheic women (79). A randomised, placebo-controlled trial in women with PCOS showed that AZD4901, a NK3R antagonist, reduced LH pulse frequency, serum LH and testosterone concentrations in women with PCOS (80). This indicates that NKB action on KNDy neurons, may be enhanced in women with PCOS and their activity could represent a target for treatment.

These conditions highlight how relative inhibition or stimulation of KNDy neurons affects the HPG axis, with detrimental effects fertility. Importantly, manipulation of these circuits could be useful in developing future therapies.

## Conclusion

LH pulsatility and the midcycle LH surge are necessary for normal follicular development and ovulation, respectively. Hypothalamic KNDy neurons release KP to stimulate GnRH neurons and induce pulsatile secretion of GnRH and subsequently gonadotrophins. Inhibition of the activity of KNDy neurons in states of stress, or over/under nutrition can result in inhibition of the reproductive axis. Manipulation of these neuronal circuits may be useful in developing future therapies for diseases caused by derangements in KNDy neuronal firing, including PCOS and functional hypogonadotrophic hypogonadism.

## Author contributions

HS, SB, MC, SM, TP, AP, OV, IA, AL, KK, and BP wrote sections of the manuscript and contributed to manuscript revision. AA contributed to revision and editing of the manuscript. All authors contributed to the article and approved the submitted version.

## Funding

All illustrations are original and were created with BioRender.com. This article presents independent research. The Section of Endocrinology and Investigative Medicine is funded by grants from the MRC, NIHR and is supported by the NIHR Biomedical Research Centre Funding Scheme and the NIHR/ Imperial Clinical Research Facility. KK is supported by NIHR Academic Clinical Fellowship Award ACF-2021-21-001 and acknowledges infrastructure support for this research from the NIHR Imperial Biomedical Research Centre (BRC). BP is supported by an MRC Clinical Research Training Fellowship (No. MR/W024144/1). AA is supported by an NIHR Clinician Scientist award (CS-2018-18-ST2-002).

## Conflict of interest

AA has undertaken consultancy work for Myovant Sciences Ltd.

The remaining authors declare that the research was conducted in the absence of any commercial or financial relationships that could be construed as a potential conflict of interest.

## Publisher’s note

All claims expressed in this article are solely those of the authors and do not necessarily represent those of their affiliated organizations, or those of the publisher, the editors and the reviewers. Any product that may be evaluated in this article, or claim that may be made by its manufacturer, is not guaranteed or endorsed by the publisher.
